# Dietary Intake of *n*-3 PUFA-Enriched Hen Eggs Changes Inflammatory Markers’ Concentration and Treg/Th17 Cells Distribution in Blood of Young Healthy Adults—A Randomised Study

**DOI:** 10.3390/nu13061851

**Published:** 2021-05-28

**Authors:** Nikolina Kolobarić, Ines Drenjančević, Anita Matić, Petar Šušnjara, Zrinka Mihaljević, Martina Mihalj

**Affiliations:** 1 Department of Physiology and Immunology, Faculty of Medicine Osijek, Josip Juraj Strossmayer University of Osijek, J. Huttlera 4, 31000 Osijek, Croatia; nbdujmusic@mefos.hr (N.K.); ines.drenjancevic@mefos.hr (I.D.); amatic@mefos.hr (A.M.); psusnjara@mefos.hr (P.Š.); zmihaljevic@mefos.hr (Z.M.); 2 Scientific Center of Excellence for Personalized Health Care, Josip Juraj Strossmayer University of Osijek, Trg Svetog Trojstva 3, 31000 Osijek, Croatia; 3 Department of Dermatology and Venereology, Osijek University Hospital, J. Huttlera 4, 31000 Osijek, Croatia

**Keywords:** fatty acids, dietary supplements, eicosanoids, inflammation, T lymphocytes

## Abstract

In the present study, we aimed to determine the effects of *n*-3 polyunsaturated acid (PUFA) supplementation (~1053 mg/per day), i.e., α-linolenic (~230 mg), eicosapentaenoic (~15 mg), and docosahexaenoic acid (~105 mg), through hen eggs, on pro- and anti-inflammatory parameters in healthy individuals (23.8 ± 2.57 years old). Here, we demonstrate differential effects of regular hen eggs (*N* = 21; W/M = 10/11) and *n*-3 PUFA-enriched hen eggs (*N* = 19; W/M = 10/9) consumption on the serum levels of lipid mediators, representation of peripheral T helper cell subsets (recently activated T-helper cells, nTreg, Th17 and non-Th17-IL-17A secreting T-helper lymphocytes) and their functional capacity for cytokine secretion. Both diets significantly altered systemic levels of pro-inflammatory and inflammation resolving lipid mediators; however, only the *n*-3 PUFAs group showed a significant shift towards anti-inflammatory prostanoids and increased levels of pro-resolving oxylipins. Both study groups showed reduced frequencies of peripheral nTreg lymphocytes and decreased rates of peripheral Th17 cells. Their functional capacity for cytokine secretion was significantly altered only in the *n*-3 PUFAs group in terms of increased transforming growth factor β-1 and reduced interleukin 6 secretion. Diet supplemented with *n*-3 PUFAs alters immune response towards inflammation resolving conditions through effects on lipid mediators and cytokine secretion by T lymphocytes in human model without underlying comorbidities.

## 1. Introduction

The finely tuned process of inflammation consists of several steps, including immune cell activation, recruitment of the cells to the site of inflammation, the release of inflammatory mediators, and increase in vascular permeability leading to resolution of the inflammatory process and healing [1]. Failure of the immune system to mitigate and efficiently resolve inflammation leads to the chronic condition of low-grade inflammation, which underlies the development of chronic diseases such as cardiovascular (CVD) and metabolic diseases [2]. Considering that, there is a certain responsibility for finding convenient tools that could improve the rate of efficient resolution in the context of reduced inflammation conditions.

In adaptive immune response to *noxis* (infection; mechanical, thermal, or chemical stimuli), several classes of CD4^+^ effector T lymphocytes are differentiated from naïve T cells, including T helper 17 lymphocytes (Th17) and the T regulatory lymphocytes (Tregs) [3,4,5]. The interplay between pro-inflammatory Th17 and immunosuppressive Tregs is of crucial importance for homeostasis and overall immunity, one being the active participant in generating inflammatory environment, and the latter being a suppressor of further pathological processes, activation, proliferation, and effectors function [3,6]. Proliferative capacity and accumulation of inflammatory T cells can be suppressed by dietary *n*-3 PUFAs intake, which leads to altered immune response through effects on lipid mediators, cytokines, and immune cell abundance [7,8].

Lipid mediators, derived from essential *n*-3 or *n*-6 polyunsaturated fatty acids (PUFAs) through cyclooxygenase (COX) and lipoxygenase (LOX) enzymatic pathways [9,10], and immune-cell-derived cytokines have significant effects on the innate and adaptive immune system [11,12,13]. Pro-inflammatory prostaglandins (PGs) and leukotrienes (LTs) are derivatives of long-chain *n*-6 PUFA-arachidonic acid (AA), while minimally inflammatory oxylipins (PG, LT) and inflammation resolving resolvins (Rvs), protectins (PDs), and maresins (MaRs) originate from long-chain *n*-3 PUFAs-α-linolenic (ALA), eicosapentaenoic (EPA) and docosahexaenoic acid (DHA) [9]. AA derivatives play a crucial role in triggering and maintaining the inflammation, while EPA and DHA derivatives have a role in terminating the inflammatory response and blocking further cell recruitment while promoting phagocytosis and tissue recovery [10,14].

Due to their involvement in inflammation and its resolution, the intake ratio between *n*-3 and *n*-6 PUFAs is of great importance for homeostasis and overall well-being [15]. There has been a shift in Western diet towards increased dietary intake of *n*-6 PUFAs and a devastating decline in *n*-3 PUFAs intake, with the ratio being 15–30:1, as opposed to ideal 2–6:1 [15,16]. As a result, the balance between the pro- and the anti-inflammatory derivatives that are synthesised from fatty acid precursors is disrupted, contributing to the pathogenesis of CVD and cancer [15,16,17,18].

There has been growing clinical end epidemiological evidence of positive effects of dietary *n*-3 PUFAs intake on the chronic inflammatory disorders, in terms of immunomodulatory, anti-inflammatory, and cardioprotective effects [19,20,21,22]. Such beneficial impacts were observed in human models, which include increased myokine irisin serum levels, elevated gene expression of sirtuins, decreased TLR4 expression, reduced LDL-cholesterol and hsCRP serum levels [19,23,24,25], while in animal models, previously reported effects included decreased levels of pro-inflammatory, and increased levels of anti-inflammatory cytokines, reduced placental oxidative stress and infarct size [26,27,28,29].

Food with biologically active properties (*n*-3 PUFA-enriched foods) can potentially provide a sufficient tool for the protection from future chronic inflammation-mediated diseases [21,30,31,32]. Recently, we have demonstrated that hen egg consumption significantly increased systemic levels of anti-inflammatory cytokine IL-10—an effect that was significantly more pronounced if the hen eggs were enriched with *n*-3 PUFAs [21]. In addition, young healthy individuals who consumed a diet containing *n*-3 PUFA-enriched hen eggs had lower levels of serum interferon gamma (INF-γ) after diet, suggesting positive effects of *n*-3 PUFA on systemic low-grade inflammation. Thus, the main objective of the present study was to address the effects of *n*-3 PUFA-enriched hen eggs consumption on the systemic levels of pro-/anti-inflammatory lipid mediators and the frequencies of peripheral immune cells in young, healthy individuals without any underlying chronic conditions. More specifically, we aimed to determine if the changes in the production of lipid mediators originating from *n*-6 and *n*-3 PUFAs, induced by functional food intake, have any effect on the peripheral T cell activation and differentiation status (Th17 and Treg).

## 2. Materials and Methods

### 2.1. Study Design and Participants

**Figure 1:**
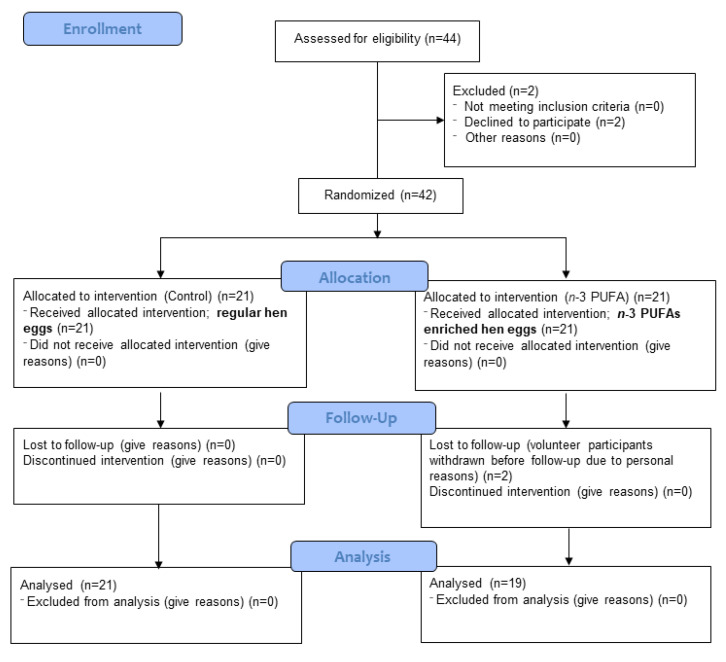
CONSORT 2010 flow diagram.

This was a randomised, double-blind, placebo-controlled study (part of ID NCT02720250 Omega-3 Fatty Acids Enriched Food and Microvascular Reactivity). The research was carried out at the Department of Physiology and Immunology, Faculty of Medicine in Osijek, Croatia. A total of 44 young and healthy volunteers were assessed for eligibility. Two volunteers decided to decline participation in the study protocol, while two participants who were enrolled in the study, failed to finish the study protocol due to personal reasons. Ultimately, 40 young healthy adults of both sexes, aged between 19–28 years old (23.8 ± 2.57 years old), participated in this study. Exclusion criteria for participants were smoking, prior history of hypertension, renal or cerebrovascular impairments, coronary artery disease, diabetes mellitus, and chronic inflammatory disorders. Volunteers who met the inclusion and exclusion criteria were included in the study by the researcher and were awarded a label indicating the project, group of respondents (“healthy”), and ordinal number. A CONSORT diagram is presented in Figure 1. A CONSORT checklist is presented as supplementary material (Supplementary Figure S1).

The study protocol lasted for three weeks and included two appointments. All study participants were instructed to eat three hard-boiled hen eggs (M commercial size) per day for the duration of the protocol (three weeks). Participants were divided into two groups: control group (*N* = 21; W/M = 10/11) consumed regular hen eggs (*n*-3 PUFAs content ~249 mg/per day), while *n*-3 PUFAs group (*N* = 19; W/M = 10/9) consumed *n*-3 PUFA-enriched hen eggs *(n*-3 PUFAs content ~1053 mg/per day). The dates of arrival of the respondents were scheduled in advance by the researcher. Eggs were divided by an unbiased associate according to the prearranged schedule of arrivals that did not contain personal data but previously assigned labels. The simple randomisation procedure was performed using a coin (letter-1 or head-2) to assign group affiliation by the unbiased associate who added the number 1 or 2 in brackets to the previously mentioned label for each individual subject, after the ordinal number, depending on which group it is: 1–a control group that consumed regular hen eggs; 2–*n*-3 PUFAs group that consumed *n*-3 PUFA-enriched eggs. Labels indicating group affiliation were known only to the associate assigning the intervention, while neither study participants nor the researcher knew to which group they belonged throughout the duration of the study. This particular associate did not participate in any of the performed analyses related to this specific study population.

Enrichment of hen eggs was executed by an associate research group from the Faculty of Agrobiotechnical Sciences Osijek, Josip Juraj Strossmayer University of Osijek, by replacing soybean oil (5%) with a mixture of fish (1.5%) and linseed (3.5%) oil in feed mixtures fed to laying hens. The fatty acid profile of the edible part of eggs used in the study was previously elaborated by our research group [21]. In short, each enriched egg (edible part ~60 g) contained approximately 351 mg of *n*-3 PUFAs in total (~230 mg of ALA, ~15 mg of EPA, ~105 mg of DHA). Participants consumed a total of 63 hard-boiled eggs during the study.

Study participants kept a diet diary in the form of 24 h recalls during study protocol, previously designed and published by our research group [33]. They were instructed to follow their usual meal schedule and to avoid taking any supplements that could alter final results, especially other *n*-3 PUFA supplements. At each appointment, the blood samples were taken for peripheral blood mononuclear cell isolation and serum collection. Between appointments, participants were contacted several times via E-mail and/or phone call to assure compliance to study protocol.

The primary outcome of the study included a change in the frequency of T lymphocytes–Tregs and Th17–after the dietary protocol, while secondary outcomes included quantification of serum levels of lipid mediators originating from *n*-6 (LTB4, PGE2) and *n*-3 fatty acids (LTB5, PGE3, RvE1), and pro- (IL17A, IL-23, IL-6) and anti-inflammatory cytokines (IL-10), growth factor (TGF-β1) and chemokine (MCP-1) secretion following PMA–ionomycin activation. Preliminary data were gathered from a total of 10 respondents after the same dietary protocols as described above (*N*(Control) = 5; *N* (*n*-3 PUFA) = 5), considering the primary outcome. The same simple randomisation procedure was performed and given the results obtained, effect and sample size were calculated before the recruitment of participants for the main study. 

This study was conducted according to the guidelines laid down in the Declaration of Helsinki, and all procedures involving research study participants were approved by the Ethical Committee of the Faculty of Medicine, University of Osijek (CLASS: 602-04/20-08/07; Reg. No.:2158-61-07-20-25). No selection bias was present in our study. All study participants signed informed consent prior to the inclusion in the study, and there was no compensation provided for their participation. Fresh eggs were delivered from the farm (Marijančanka d.o.o.) to the laboratory once a week and distributed to participants entering the protocol within 7 days.

### 2.2. Anthropometry and Laboratory Testing 

Venous blood samples were taken after an overnight fast on the first and last day of the protocol. Samples were analysed for full blood cell count, plasma electrolytes (sodium, potassium, calcium), iron, transferrin, creatinine, urea, fasting blood glucose, high-sensitivity C-reactive protein (hsCRP), and fasting lipid profile (total cholesterol, low-density lipoprotein cholesterol, high-density lipoprotein cholesterol, triglycerides) using standard laboratory methods and operating protocols at the Clinical Department of Laboratory Diagnostics, University Hospital Osijek. All analyses were performed on Olympus instrument using IVD certified reagents and protocols provided by the manufacturer. Sodium, potassium, and calcium were measured by potentiometry; hsCRP, transferrin, and ferritin (iron) were measured using immunoturbidimetric assays; blood cell count, haematocrit levels, red cell indices (MCV, MCH, MCHC), RDW-CV, and MPV were evaluated by impedance spectroscopy; other parameters were measured by spectrophotometry. The lipid profile was measured directly.

Body mass index (BMI) was calculated according to the standard formula (BMI = body mass/height in m^2^) using body mass (kg) and height (m) measures obtained at each appointment by the researcher.

### 2.3. Peripheral Blood Mononuclear Cells (PBMCs)

#### 2.3.1. Isolation from Whole Blood

Venous blood samples were collected in 10 mL vacutainer tubes containing EDTA and processed within three hours of collection. Refrigerated reagents and buffers used in isolation were warmed up to room temperature (RT, ~20–25 °C) prior to isolation. Collected whole blood was diluted with pre-prepared 1× phosphate-buffered saline (PBS) at 1:1 ratio and carefully layered on Ficoll-Paque^®^ PLUS centrifugation media (GE Healthcare Bio-Sciences AB, Uppsala, Sweden) without mixing the layers. Following, samples were centrifuged for 25 min at 800 G with breaks off, at RT (Rotina 380, Hettich GmbH & Co. KG, Tuttlingen, Germany). The layer containing mononuclear cells was collected and rinsed twice with 1× PBS. Cell numbers were determined by staining the cells with 0.4% Trypan blue solution (Sigma-Aldrich, Merck KGaA, Darmstadt, Germany) and using a Bürker-Türk counting chamber. (Accessed date 20 June 2019; Modification of protocol available at https://www.cptp.inserm.fr/wp-content/uploads/2018/01/PBMC-isolation-and-cryopreservation.pdf).

#### 2.3.2. Cell Culture

PBMCs were cultured in RPMI-1640 media with L-glutamine (Sigma-Aldrich), supplemented with the addition of fetal bovine serum (10%) (FBS; Sigma-Aldrich) and penicillin–streptomycin antibiotic (1%) (Capricorn Scientific GmbH, Ebsdorfergrund, Germany). Cell suspensions were stored in 24-well plates and placed in an incubator (Shel Lab, CO_2_ Series, Sheldon manufacturing Inc, Cornelius, OR, USA) under the following conditions: ~37 °C, 5% CO_2_, >80% humidity level for 24 h before any further proceedings. 

#### 2.3.3. Cryopreservation and Thawing 

For the purpose of cryopreservation, dimethyl sulfoxide (DMSO; Supelco, Merck KGaA, Darmstadt, Germany) and FBS were used at a 1:9 ratio. Additionally, for optimal cell preservation, the cryovials were stacked in Mr. Frosty freezing container containing isopropyl alcohol and placed in a −80 °C freezer for at least 24 h.

Thawing of samples was carried out with FBS/antibiotics supplemented RPMI-1640 culture media preheated to ~37 °C. Prior to adding the media, cryovials containing cells were carefully dipped into a water bath (~37 °C) for roughly 1 min and then transferred to larger tubes. The preheated medium was pipetted onto the cells in a drip mode to avoid osmotic shock and the samples were centrifuged. After two additional washing steps, cells were resuspended in a fresh medium, transferred to 24-well plates, and kept in an incubator for 24 h (~37 °C, 5% CO_2_, >80% humidity).

#### 2.3.4. Cell Viability

Analyses to identify cell viability and exclude possible bias induced by nonspecific staining of dead/dying cells in our samples included (a) staining cells with 0.4% Trypan blue solution and counting live cells in the Bürker-Türk chamber under a light microscope and (b) staining cells with fixable viability dye (FVD) eFluor™ 780 (eBioscience^TM^, Invitrogen by Thermo Fisher Scientific, Waltham, MA, USA), which is detectable on a flow cytometer upon excitation with 633 nm red laser. Samples included in the final analysis and calculations had cell viability of ≥80%.

#### 2.3.5. Magnetic Cell Sorting 

After thawing the samples and adjusting the cell numbers to 1.2 × 10^7^, CD4^+^ T cells were separated using negative magnetic selection via commercially available magnetic beads (MagniSort^TM^ Human CD4^+^ T cell, Enrichment kit; Invitrogen by Thermo Fisher Scientific, Waltham, MA, USA), and following the protocol provided by the manufacturer (Accessed date 11 May 2020; Protocol available at https://assets.thermofisher.com/TFS-Assets/LSG/manuals/8804-6811.pdf). Negatively selected cells were prepared for the activation.

#### 2.3.6. Activation of CD4^+^ T Lymphocytes

In order to activate CD4^+^ T cells and promote cytokine production, magnetically sorted cells were shortly (4 h) stimulated by phorbol 12-myristate 13-acetate (PMA) and ionomycin. CD4 T cell activation was carried out in 24-well plates (4 hrs, ~37 °C, 5% CO_2_, >80% humidity level) with a commercially available cell stimulation cocktail (500×; eBioscience^TM^, Invitrogen by Thermo Fisher Scientific, Waltham, MA, USA) at a final concentration of 2 µL/mL (Accessed date 12 May 2020; full protocol available at https://assets.thermofisher.com/TFS-Assets/LSG/manuals/00-4970.pdf). 

Calcium Chloride (CaCl_2_) was added to the stimulation media to provoke a long-lasting intracellular calcium signalling that would evoke a cellular response (5 µL/mL final concentration).

In order to prevent cytokine secretion and allow assessment of IL-17 producing cells by flow cytometry (intracellular IL-17 staining, detailed protocol given in Section 2.4), Brefeldin A solution (1000×; eBioscience^TM^, Invitrogen by Thermo Fisher Scientific, Waltham, MA, USA) was used as an inhibitor of protein transport to Golgi apparatus with resulting accumulation of proteins in the endoplasmic reticulum (Accessed date 12 May 2020; 3 µL/mL final concentration; available at https://assets.thermofisher.com/TFS-Assets/LSG/manuals/00-4506.pdf). 

Cell stimulation cocktail, Brefeldin A and CaCl_2_ were added together and at the same time to the cell suspension. After completion of four-hour incubation, 200 µL of 0.1 M EDTA was added and incubated for 15 min at RT in order to stop the reaction.

### 2.4. Flow Cytometry 

Frequencies of CD4^+^Foxp3^+^ regulatory T lymphocytes and CD4^+^IL-17A^+^ T helper lymphocytes among isolated peripheral blood mononuclear cells were determined by the flow cytometry method. Sample preparation and staining protocols for intracellular antigens for flow cytometry were modified versions of recommended protocols (Accessed date 13 May 2020; available at www.thermofisher.com). For intracellular staining, a Foxp3 transcription factor staining buffer set was used (eBioscience^TM^, Invitrogen by Thermo Fisher Scientific, Waltham, MA, USA). In short, prior to cell surface staining, fixation/permeabilisation, and intracellular/nuclear staining steps, dead cells were irreversibly labelled with previously mentioned FVD, and nonspecific antibody capturing by Fc receptors was blocked by the addition of human Fc-blocking reagent (BD Pharmigen^TM^, BD Biosciences, Becton, Dickinson and Company, Franklin Lakes, NJ, USA). After incubation at RT, staining with appropriate antibody mixture was carried out depending on the cell subset of interest. Along with careful sample preparation and optimisation of staining protocols, single-stain, fluorescence minus one (FMO), unstained and negative controls were included in our experiments in order to reliably distinguish positive cells from background/negative staining and nonspecific effects.

The fluorescence compensation matrix for multicolour flow cytometry analysis was calculated using BD^TM^ CompBeads Anti-Mouse Ig, κ/Negative Control Compensation Particle Set (BD Biosciences, Becton, Dickinson and Company, Franklin Lakes, NJ, USA). Measurements of stained samples were carried out by BD FACSCanto II cytometer (FACSCanto II, Becton Dickinson, San Jose, CA, USA) equipped with blue Argon 488 nm and Red HeNe 633 nm laser lines. Data analysis and visualisation were performed using the FlowLogic software (Inivai Technologies, Mentone, Australia).

#### 2.4.1. Regulatory T Lymphocytes (Tregs) 

To assess the frequencies of Treg cells among PBMCs, the following mouse anti-human antibodies mixture for the cell surface staining was used: CD3 FITC (clone: OKT3, eBioscience^TM^, Affymetrix by Thermo Fisher Scientific, CA, USA), CD4 PerCP-eFluor^TM^ 710 (clone: SK3, eBioscience^TM^), CD127 PE-Cy7 (clone: eBioRDR5, eBioscience^TM^), CD25 APC (clone: BC96, eBioscience^TM^); while Foxp3 PE (clone: 235A/E7, eBioscience^TM^) antibody was used for intracellular staining of cells. The optimal antibody concentration was determined by antibody titration experiment on 1 × 10^6^ PBMCs and based on stain-index calculations. 

#### 2.4.2. Helper T Lymphocytes (Th17)

Th17 lymphocytes’ rate among total peripheral blood CD4 lymphocytes were determined using PMA–ionomycin-activated CD4 cells and the following mouse anti-human antibodies mixture: CD3 PerCP-eFluor^TM^ 710 (clone: SK7, eBioscience^TM^, Affymetrix by Thermo Fisher Scientific, CA, USA), CD4 PE-Cy7 (clone: SK3, eBioscience^TM^), CD196 APC (clone: R6H1, eBioscience^TM^) for cell surface antigens, and RORɣt PE (clone: AFKJS-9, eBioscience^TM^) and IL-17A FITC (clone: eBio64DEC17, eBioscience^TM^) for intracellular antigens.

### 2.5. Luminex Assay 

#### 2.5.1. Collection of Supernatants from PMA–Ionomycin-Treated PBMC Cell Cultures 

After thawing, the PBMCs were allowed to rest and recover overnight in culture media (~37 °C, 5% CO_2_, >80% humidity level). Before proceeding to the next step, cells were counted, and the number of cells was adjusted to 300,000 cells per 200 µL of stimulation media for each sample. Stimulation media consisted of supplemented RPMI-1640 culture media, PMA–ionomycin. and CaCl_2_ at previously mentioned final concentrations (Section 2.3). PBMC activation was carried out in 96-well plates (4 h, ~37 °C, 5% CO_2_, >80% humidity level). Following the 4-h incubation period, the collected supernatant was stored at −80° C until analysis.

#### 2.5.2. Multiplex and Simplex Protein Quantitation of Pro- and Anti-Inflammatory Cytokines, Transforming Growth Factor, and Chemokine Supernatant Concentrations

Concentrations of pro- and anti-inflammatory cytokines, including interleukin 17A (IL-17A), interleukin 23 (IL-23), interleukin 6 (IL-6), interleukin 10 (IL-10); and transforming growth factor-beta 1 (TGF-β1); and the levels of monocyte chemoattractant protein-1 (MCP-1) chemokine were measured using the Invitrogen ProcartaPlex antibody-based, magnetic bead reagent kits (Invitrogen by Thermo Fisher Scientific, Waltham, MA, USA) and panels for multiplex and simplex protein quantitation on the Luminex 200 instrument platform (Luminex Corp., Austin, TX, USA). Measurements were performed in the Laboratory of Molecular and HLA Diagnostics of the University Hospital Osijek (Osijek, Croatia). Data were analysed by using ProcartaPlex Analyst free software (eBioscience, Affymetrix by Thermo Fisher Scientific, Waltham, MA, USA) and are expressed as a concentration in picograms per millilitre.

### 2.6. ELISA 

Serum concentrations of leukotriene B4 (LTB4), leukotriene B5 (LTB5) (Cusabio, Houston, TX, USA), prostaglandin E2 (PGE2), prostaglandin E3 (PGE3) (MyBioSource, MyBioSource Inc., San Diego, CA, USA), resolvin E1 (RvE1) (MyBioSource, MyBioSource Inc., CA, USA) were measured by commercially available enzyme-linked immunosorbent assay (ELISA) kits on compact absorbance reader for 96-well microplates (BioRad PR 3100 TSC, Bio-Rad Laboratories, CA, USA).

### 2.7. Statistical Analysis

Statistical analyses were performed using Microsoft Excel 2016 (Microsoft Office 365, Microsoft Corporation, Redmond, WA, USA), Graph Pad Prism v6.01 (GraphPad Software, San Diego, CA, USA), and SigmaPlot v11 (Systat Software, Inc., Chicago, IL, USA) software. Cohen’s d (∆/SD) effect size was determined according to the primary outcome of the study—change in the frequency of T lymphocytes, Tregs, and Th17. GPower v3.1.9.7 (Heinrich Heine University Düsseldorf, Düsseldorf, Germany) was used for sample size calculation. 

During optimisation of the protocol and design of the research, a pilot study was conducted with a total of 10 respondents. Effect size (Cohen d, ∆/SD) required for a statistical strength of 80% with bilateral α = 0.05, paired *t*-test, before and after the dietary protocol is 1.1 for the Treg lymphocyte population and requires a sample of at least 13 subjects per group (GPower v3.1.9.7). For the estimated, expected arithmetic means of the frequency of Foxp3^+^ Treg lymphocytes in the peripheral blood of healthy individuals before (16.8%) and after the dietary protocol (9.2%), with corresponding standard deviations of 6.1% and 7.2%, the difference the arithmetic mean is 9% which corresponds to a biologically large effect. The effect size required for a statistical strength of 80% with bilateral α = 0.05, paired *t*-test, before and after the dietary protocol is 0.9 for the Th17 lymphocyte population and requires a sample of a minimum of 19 subjects per group. For estimated, expected arithmetic means of the frequency of IL-17A^+^ Th17 lymphocytes in the peripheral blood of healthy individuals before (0.72%) and after the dietary protocol (0.49%), with corresponding standard deviations of 0.29% and 0.19%, the difference of arithmetic means is 11%, which corresponds to a biologically large effect. 

The normality of distributions was tested by Shapiro–Wilk test. Data are presented as average ± standard deviation (SD). Student’s *t*-test and Mann–Whitney tests were used for group comparisons, while Paired *t*-test and Wilcoxon rank-sum tests were used to test the differences between the measurements within a group. Correlations between paired datasets were determined by the Spearman rank test. Two-tailed *p* < 0.05 was considered significant. 

## 3. Results

### 3.1. Anthropometric and Biochemical Characteristics of Study Population

The general and biochemical characteristics of the individuals enrolled to participate in this study are shown in Table 1. There were no significant differences in the age between the control (23.8 ± 2.79 years old) and the *n*-3 PUFAs group (23.8 ± 2.34 years old). Both sexes were equally represented in the study groups. The average BMI of both control (24.2 ± 3.01 kg/m^2^) and *n*-3 PUFAs group (22.72 ± 3.53 kg/m^2^) was within the normal range according to the WHO criteria for European population weight classification (18.5–24.9 kg/m^2^). Based on the medical history and the performed laboratory tests, all participants were healthy. Their full blood count, serum electrolytes, fasting blood glucose levels and hsCRP were within the normal range. Baseline total cholesterol and LDL-cholesterol levels were found to be slightly elevated in the control group, compared to the *n*-3 PUFAs group (*p* = 0.012 and *p* = 0.037, respectively), and above recommended upper limits of the population, while all other biochemical parameters were within the normal reference range for the general population. In a previous paper by Stupin et al. (2020) [21] including the same study population and protocol, we reported that levels of hsCRP were not significantly changed by consumption of regular or *n*-3 PUFA-enriched eggs, compared to respective baseline measurements, nor was there a difference in hsCRP between the groups. In addition, there were no significant differences in serum total cholesterol, triglycerides, LDL cholesterol, and HDL cholesterol concentrations after regular or *n*-3 PUFA-enriched eggs consumption, compared to respective baseline values. According to the anthropometric, biochemical, and hemodynamic parameters, body composition and body fluid status were all evaluated in the previously published paper by Stupin et al. (2020.); our study groups were uniformly distributed [21].

### 3.2. n-3 PUFA Supplementation Changes the Ratio of Pro- and Anti-Inflammatory Lipid Mediators Originating from n-6 (AA) and n-3 (EPA) Fatty Acids

**Figure 2:**
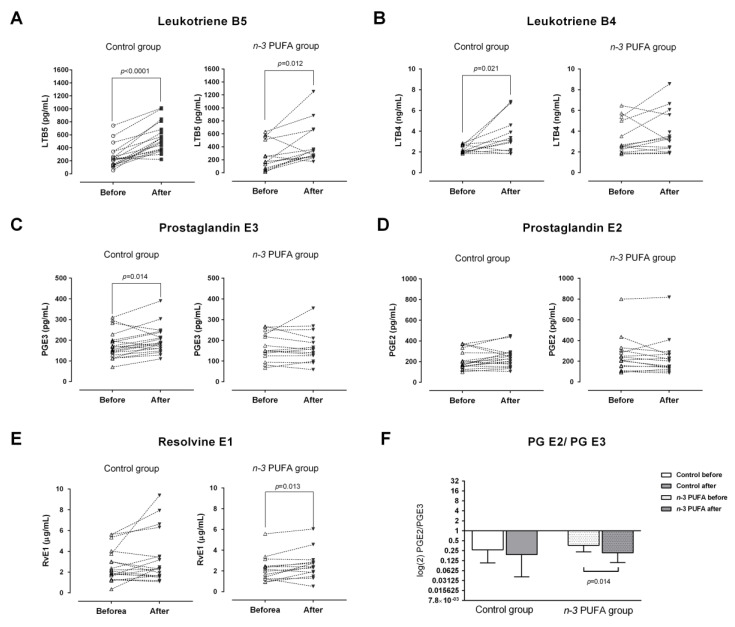
Effects of regular (control group) and *n*-3 polyunsaturated acid (PUFA)-enriched hen egg (*n*-3 PUFA group) consumption on the serum concentrations of pro-inflammatory leukotriene B4 (**B**) and prostaglandin E2 (**D**); inflammation resolving leukotriene B5 (**A**), prostaglandin E3 (**C**) and resolvin E (**E**) lipid mediators originating from *n*-6 (arachidonic acid; AA) and *n*-3 (eicosapentaenoic acid; EPA) fatty acids. The ratio between prostaglandin E2 and prostaglandin E3 is shown at Panel (**F**). PUFA—polyunsaturated fatty acid; LTB4 – leukotriene B4; LTB5—leukotriene B5; PGE2 - prostaglandin E2; PGE3—prostaglandin E3; RvE1—resolvin E1. Paired *t*-test; significance level *p* < 0.05; before protocol vs. after protocol.

Serum concentrations of pro-inflammatory eicosanoids (LTB4 and PGE2) from AA, and inflammation resolving oxylipins (LTB5 and PGE3) and resolvins (RvE1) from EPA and DHA before and after the dietary protocols are shown in Figure 2. 

LTB4 and PGE3 serum levels were significantly increased in the control group after three-week consumption of regular hen eggs (*p* = 0.021 and *p* = 0.014, respectively, Figure 2B,C), while their levels remained unchanged in the *n*-3 PUFAs group. Average serum concentrations of LTB5 at the end of the dietary protocol were significantly increased in both groups, compared to their respective baseline levels (*p* < 0.0001 and *p* = 0.012, respectively; Figure 2A). Serum level of RvE1 was significantly increased in the *n*-3 PUFAs group after the three-week consumption of *n*-3 PUFA-enriched hen eggs (*p* = 0.013; Figure 2E). Serum concentrations of PGE2 were not significantly affected by any of the dietary protocols (Figure 2D). To address the proportions of pro-inflammatory and anti-inflammatory lipid mediators, prostaglandin E2/E3 and leukotriene B4/B5 ratios were calculated and compared across the measurements. These results showed a significant decrease in prostaglandin E2/E3 ratio following *n*-3 PUFA dietary protocol (*p* = 0.014; Figure 2F), while the leukotriene B4/B5 ratio remained unchanged in both groups.

### 3.3. PBMC-Derived Cytokines Following PMA–Ionomycin Activation 

**Figure 3:**
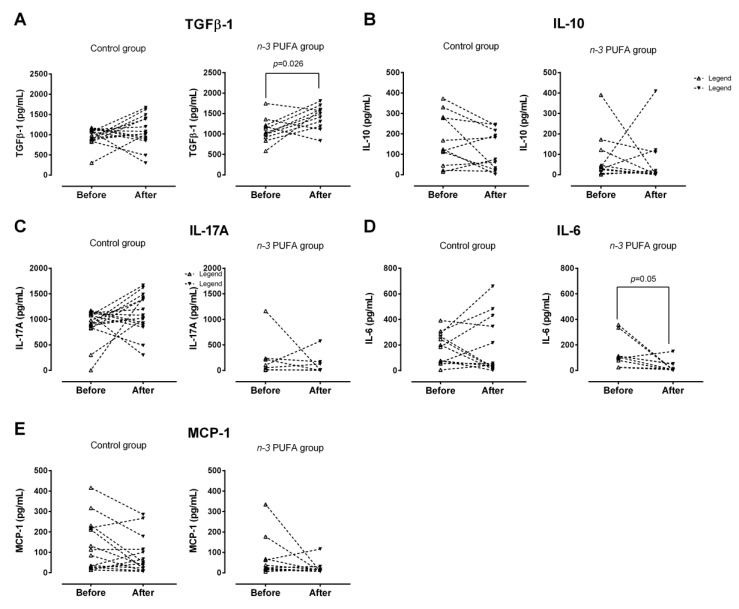
Levels of pro-/anti-inflammatory cytokines and chemokines secreted by PBMCs upon PMA/ionomycin stimulation in young individuals following regular (control Group) or *n*-3 polyunsaturated acid (PUFA)-enriched hen egg (*n*-3 PUFA group) consumption. There was a significant increase in TGFβ-1 level in *n*-3 PUFA group (**A**), while no significant changes were found for IL-10 (**B**), IL-17A (**C**), IL-6 (**D**) or MCP-1 (**E**). PBMC—peripheral blood mononuclear cells; TGFβ-1—Transforming Growth Factor Beta-1; IL-10—Interleukin 10; IL-17A—Interleukin 17A; IL-6—Interleukin 6; MCP-1—Monocyte Chemoattractant Protein-1. Paired *t*-test; significance level *p* < 0.05; before protocol vs. after protocol.

Concentrations of anti-/pro-inflammatory cytokines (IL-10, IL-6, IL-23, IL-17A, and TGF-β1) and MCP-1 chemokine in both control and experimental group, before and after dietary protocols were determined in supernatants from PBMC cell cultures upon PMA/ionomycin stimulation (Figure 3).

TGF-β1 production by peripheral blood mononuclear cells following *n*-3 PUFA dietary protocol was significantly increased (*p* = 0.026; Figure 3A), while IL-6 production was significantly decreased (*p* = 0.05; Figure 3D), compared to the respective baseline levels. In addition, end-point TGF-β1 levels were significantly lower in the control group, compared with the end-point levels measured in the *n*-3 PUFAs group (*p* = 0.004; Figure 3A). Target cytokine and chemokine production by PBMC was unaffected by the consumption of regular hen eggs (control group, Figure 3A–E). Furthermore, the intergroup analysis revealed significant differences in baseline and end-point IL-17A production, namely, in the control group, levels of IL-17 secreted by PBMC upon PMA/ionomycin stimulation were significantly higher both prior and after consumption of regular hen eggs, compared to *n*-3 PUFAs group (*p* < 0.001 and *p* = 0.001, respectively; Figure 3C).

### 3.4. Frequencies/Abundance of Peripheral Blood Treg and Th17 Lymphocytes Are Reduced Following Dietary Protocols

**Figure 4:**
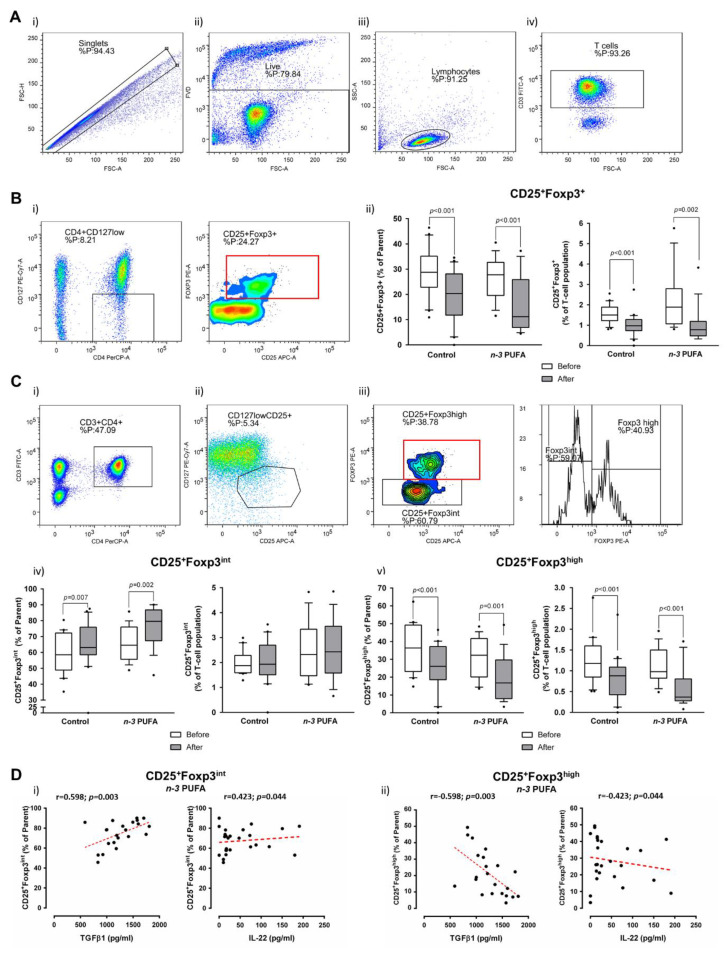
Effects of regular (control group) and *n*-3 polyunsaturated acid (PUFA)-enriched hen egg (*n*-3 PUFAs group) consumption on the frequency of peripheral regulatory T cells (Treg) in healthy young individuals. (**A**) shows representative dot plots illustrating gating strategy, including exclusion of doublets using forward scatter area (FSC-A) versus forward scatter width (FSC-W) analysis (A-i), gating on live cells negative for amine-reactive fixable viability dye (A-ii), lymphocytes (A-iii) and CD3^+^ T cells (A-iv). First, total CD25 and Foxp3 expressing T cells among CD4^+^CD127^low^ population were analysed (**B**). B-i shows representative gating strategy, while the relative frequencies are presented as box-and-whisker plots at B-ii. Next, we have analysed CD25^+^CD127^low^ subpopulation (C-ii) of T helper lymphocytes (CD3^+^CD4^+^) (C-i) for the expression of Foxp3 transcription factor (C-iii). C-i/ii/iii shows representative gating strategy, while the relative frequencies of CD25^+^Foxp3^int^ and CD25^+^Foxp3^high^ are presented as box-and-whisker plots at (C-iv) and C-v, respectively. Based on the Foxp3 expression (**B**,**C**), two T cell subpopulations were identified within CD4^+^CD127^−^CD25^+^ T cell pool: CD4^+^CD127^-^CD25^+^Foxp3^int^ recently activated T helper cells (Ci–iv); and CD4^+^CD127^−^CD25^+^Foxp3^high^ subpopulation corresponding to regulatory T cells (Ci–iii, v). (**D**) shows differential correlation of Foxp3-expressing subpopulations with TGFβ-1 (secreted upon PBMC stimulation) and serum levels of IL-22. PUFA—polyunsaturated fatty acid; TGFβ-1—Transforming Growth Factor Beta-1; PBMC—peripheral blood mononuclear cells; IL-22 - Interleukin 22. Paired *t*-test, before protocol vs. after protocol; *p* < 0.05 was considered significant; r—Spearman correlation coefficient.

Both dietary protocols resulted in significant decrease of CD25/Foxp3-expressing peripheral blood lymphocytes within CD4^+^CD127^+^ subpopulation (*p* < 0.001; Figure 4A,B). The same differences were observed when frequencies of these cells (CD25^+^Foxp3^+^) were compared within the total peripheral T helper pool (*p* < 0.001 and *p* = 0.002 for the control group and *n*-3 PUFAs group, respectively; Figure 4A,B). The observed decrease was 1.5-fold in the control group and 1.6-fold in the *n*-3 PUFAs group. Additional analysis of Foxp3 expression in T helper cells showed that the observed differences were due to the significant reduction of CD4^+^CD25^+^Foxp3^high^ subpopulation corresponding to ‘real’ regulatory T cells (nTreg) in both groups (Figure 4C,D), while the observed increase in recently activated CD4^+^CD25^+^Foxp3^int^ T cell frequencies represents a relative increase since their frequencies within the total T cell pool remained unchanged (Figure 4Civ). There were no significant differences in these subpopulations of cells between the groups, neither prior to entering the dietary protocols nor at the end of the protocols. An additional finding of this study was that the rates of recently activated T cells were positively associated, while the % of nTreg was inversely related to serum IL-22 levels in the *n*-3 PUFAs group (Figure 4D).

**Figure 5:**
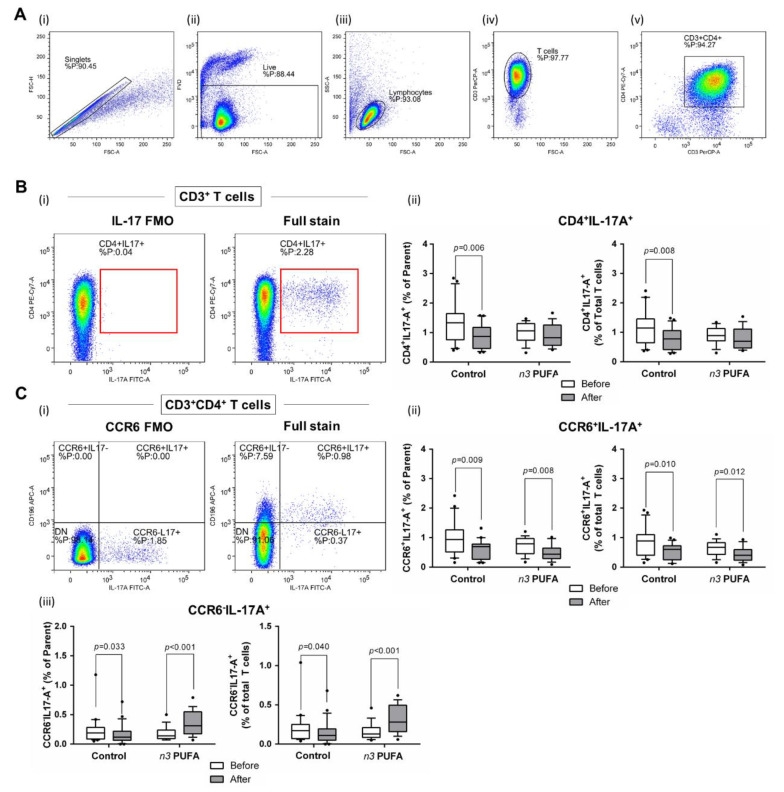
Effects of regular (control group) and *n*-3 polyunsaturated acid (PUFA)-enriched hen egg (*n*-3 PUFAs group) consumption on the representation of peripheral T helper cell subpopulations in healthy young individuals: (**A**) shows representative dot plots illustrating gating strategy, including exclusion of doublets using forward scatter area (FSC-A) versus forward scatter width (FSC-W) analysis (A-i), gating on live cells negative for amine-reactive fixable viability dye (A-ii), lymphocytes (A-iii), CD3^+^ T cells (A-iv), and CD3^+^CD4^+^ T cells (A-v). T helper cells were subsequently analysed for IL-17A (**B**) and CD196/CCR6 expression (**C**). First; all IL-17 secreting T helper cells were analysed (**B**) where the gate on IL-17^+^ T cells was defined using fluorescence minus one (FMO) control (B-i) and the relative frequencies of CD4^+^IL-17^+^ T helper cells are presented as box-and-whisker plots at (B-ii). The population of IL-17 secreting T helper cells was further analyse s for CD196/CCR6 expression (C-i), hence two subpopulations were identified—CD4^+^CD196^+^IL-17^+^ corresponding to Th17 cells (C-ii) and CD4^+^CD196^-^IL-17^+^ non-Th17 cells accounting for other T helper subpopulations with the capacity to secrete IL-17 (C-iii). There was a significant decrease in Th17 cell frequency in both groups following dietary protocol. Frequency of non-Th17 cells was significantly reduced in control group, while the same T cell subpopulation was increased in the *n*-3 PUFAs group after dietary protocol. Paired *t*-test, before protocol vs. after protocol; *p* < 0.05 was considered significant.

In the present study, a significant reduction in the frequency of total IL-17 secreting peripheral T helper cells was observed at the end of the protocol in the control group (*p* = 0.008; Figure 5Bi,ii). These cells were further immunephenotyped and, based on their CCR6 expression, subdivided to Th17 (CCR6^+^IL-17^+^) and non-Th17 (CCR6^-^IL-17^+^) T helper cells, with the latter corresponding mostly to IL-17-secreting Th1 and Th2 T helper cells. This further revealed that the frequencies of Th17 cells were significantly reduced at the end of both dietary protocols (*p* = 0.009 and *p* = 0.008 in the case of the control group and *n*-3 PUFAs group, respectively; Figure 5Ci,ii). Interestingly, in the case of CCR6^-^IL-17^+^ T cells (non-Th17 cells), the control group had significantly reduced frequency of these cells (*p =* 0.033, Figure 5Ciii), while the subjects from the *n-3* PUFAs group had significantly increased frequency of the same T cell subpopulation (*p* < 0.001, Figure 5Ci).

### 3.5. Correlation Analysis

Correlation analysis was performed to assess relationships between biochemical parameters (hsCRP, fasting lipid profile), serum concentrations of eicosanoids and resolvins, PBMC-derived cytokines and chemokines, and peripheral Treg and Th17 lymphocyte frequencies.

In the control group, rates of peripheral blood CD25/Foxp3-expressing lymphocytes were positively associated with the rates of peripheral blood IL-17 producing CD4 T cell subset (*r* = 0.326; *p* = 0.035) and inversely associated with the serum fasting cholesterol levels (*r* = −0.332; *p* = 0.036) and BMI (*r* = −0.441; *p* = 0.0036). However, after further identification of nTreg (CD25^+^Foxp3^high^) and recently activated T cells (CD25^+^Foxp3^int^) among CD25/Foxp3-expresing T helper cells, associations with CD4^+^IL-17^+^ T cell pool were lost. Interestingly, the BMI status was still inversely related to nTreg (*r* = −0.535; *p* < 0.0001) but positively associated to the rates of recently activated T helper cells (*r* = 0.399; *p* = 0.009). There was a positive correlation between prostaglandin E2 and E3 serum concentrations (*r* = 0.819; *p* = 0.0002), while there was a significant negative correlation found between hsCRP level and prostaglandin E2/E3 ratio (*r* = −0.438; *p* = 0.006).

In the *n*-3 PUFAs group, peripheral nTreg lymphocytes were negatively associated with non-Th17 IL-17A secreting T helper cells (CCR6^-^IL-17^+^; *r* = −0.613, *p* < 0.0001) and PBMC-derived TGFβ-1 (*r* = −0.616, *p* = 0.002), while recently activated T helper cells were positively associated with CCR6^-^IL-17^+^ (*r* = −0.591, *p* = 0.0003) and PBMC-derived TGFβ-1 (*r* = −0.522, *p* = 0.012). Interestingly, rates of peripheral non-Th17 lymphocytes were positively associated with PBMC-derived IL-17A levels (*r* = 0.456, *p* = 0.032), while there was no association to ‘real’ Th17 cells. Leukotriene B5 serum concentrations negatively correlated with concentrations of PBMC-derived IL-17A (*r* = −0.743, *p* = 0.029). HDL-cholesterol levels negatively correlated with hsCRP (*r* = −0.408, *p* = 0.042).

## 4. Discussion

Beneficial effects of *n*-3 PUFA supplementation are well documented in the literature, but these studies usually involved *n*-3 or *n*-6 PUFAs addition to various cell cultures [9,34,35] or, in the case of human studies, oil capsules were mostly given to patients with certain comorbidities [36,37,38]. In the present study, we aimed to determine the effects of *n*-3 PUFA supplementation, i.e., ALA, EPA, and DHA, through functional foodstuffs, on pro- and anti-inflammatory parameters in young healthy individuals. We previously established that this particular study population normally consumes extremely low amounts of *n*-3 PUFA-rich food such as fish and nuts [33]; therefore, the main effect of *n*-3 PUFAs in the present study came from the consumption of *n*-3 PUFA-enriched hen eggs. Assessment of serum fatty acid profile before and after finishing respective dietary protocols confirmed excellent compliance and significant changes to the fatty acid composition and *n*-6/*n*-3 PUFAs ratio [21]. The salient findings of the present study are differential effects of the regular hen egg and *n*-3 PUFA-enriched hen egg consumption on the serum levels of lipid mediators, representation of peripheral T helper cell subsets (recently activated T helper cells, nTreg, Th17, and non-Th17 IL-17A secreting T helper lymphocytes), and their functional capacity for cytokine secretion. Both diets significantly altered systemic levels of pro-inflammatory, and inflammation resolving lipid mediators; however, only the *n*-3 PUFAs group showed a significant shift towards anti-inflammatory prostanoids and increased levels of pro-resolvins. Both study groups showed reduced frequencies of peripheral nTreg lymphocytes and decreased rates of peripheral Th17 cells. Their functional capacity for cytokine secretion was significantly altered only in the *n*-3 PUFAs group in terms of increased TGFβ-1 and reduced IL-6 secretion.

Inhibitory effects of supplemental ALA and DHA on the COX pathway were previously documented in human umbilical vein endothelial cells (HUVECs) [9] and bovine aortic endothelial cells (BAECs) [34,35]. In addition, Araujo et al. (2019) [9] failed to find a significant association between increased EPA administration and production of PGE3 in HUVECs, suggesting ALA/DHA-specific effects on the COX pathway. A possible explanation was given by Malkowski et al. (2001) [39], who described decreased flexibility of EPA when bound to COX activity site due to an additional double bond which results in low oxygenation and enzymatic conversion to PGE3. This could also explain the results of the present study in which we were not able to prove significant effects of *n*-3 PUFAs supplementation through functional food on systemic levels of both pro- and anti-inflammatory (PGE2 and PGE3, respectively) lipid metabolites derived via the COX pathway. Even though there were no significant changes observed in PGE2 and PGE3 serum concentrations individually, there was a significant decrease in endpoint PGE2/E3 serum concentration ratio in the *n*-3 PUFAs group, compared to baseline ratio, which indicates a slight shift in favor of anti-inflammatory metabolites. 

Further finding of this study were significantly increased serum concentrations of inflammation resolving five-series of leukotrienes/LTB5 in both groups at the end of dietary protocols and increased levels of pro-inflammatory four-series of leukotrienes/LTB4 in the control group which indicates that the activity of the LOX pathway is preferred over the production of COX pathway metabolites from EPA. It has been previously shown that supplemental DHA increases the production of LOX pathway metabolites and resolvins in HUVECs [9]. This information and the fact that DHA can be retro-converted to EPA following supplementation [40,41] support our finding of increased production of RvE1 in the *n*-3 PUFAs group following diet protocol. Both E- and D-series resolvins have a crucial role in response to acute inflammation [42], especially in allergic response and asthma [43,44], and neuroinflammation [14,45], and exhibit therapeutic potential for the treatment of inflammatory bowel disease [46].

Examined lipid mediators and cytokines (secreted predominantly by T cells) act synergistically to constrain inflammation and to promote resolution after pathogen elimination, predominantly through refining T cell functions [11,12,13,43]. Cytokines act as signalling molecules and humoral regulators of cell activation, differentiation, proliferation, and cytokine production, including chemokines, interleukins (IL), growth factors, and interferons (IF) [12,13,47]. T lymphocytes manage cell immunity through activation of phagocytes and by promoting the destruction of pathogens and infected cells while also producing cytokines with specific effects on the other immune cells [3,11]. Cytokine secretion by T lymphocytes was significantly altered by *n*-3 PUFAs in our study towards reduced inflammation, as further described.

Haworth et al. (2008) [43] reported that increased RvE1 decreases IL-17A concentration by ~70% in an animal model. This effect, however, could not be observed in our study population. As elaborated in the Results Section, in our cohort of healthy young adults, a significant difference was observed regarding IL-17A levels in the control group, both prior and after the protocol, when compared to the *n*-3 PUFAs group. Therefore, we performed series of correlation analyses between the IL-17A levels and anthropometric/biochemical parameters which could not explain the initial differences in IL-17A production between the groups. Ongoing acute infection or underlying immune-mediated inflammatory disorders were eliminated based on the medical records and blood tests. However, a significant positive association was observed between RvE1 and systemic levels of IL-10, IL-22, IL-6, and IL-9 only in the *n*-3 PUFAs group. In addition, the functional capacity of lymphocytes to produce IL-17A was inversely related to systemic levels of pro-resolving LTB5 in the *n*-3 PUFAs group, which is, alongside RvE1, also an EPA-derived mediator. Furthermore, the inhibitory effect of RvE1 on IL-6 production was previously reported in human neutrophil cell lines [48], and IL-6 was another cytokine measured in our study, namely, IL-6 secretion by lymphocytes upon PMA/ionomycin activation was significantly decreased in *n*-3 PUFAs group at the end of the dietary protocol. 

Above mentioned pro-inflammatory cytokines, alongside IL-23, have a detrimental role in the differentiation and survival of Th17 cells as well as in mediating Th17 effector functions. In addition, previous research suggests that they can be downregulated by increased RvE1 production [43], which we have partly confirmed by the observation that IL-6 production by peripheral lymphocytes was reduced, in parallel with increased systemic levels of RvE1 in the *n*-3 PUFAs group. In addition, there was decreased prevalence of peripheral Th17 lymphocytes following hen eggs consumption, independently of their *n*-3 PUFA enrichment. 

TGFβ-1 and IL-10 cytokines have a central role in maintaining the immune balance by limiting immune reactions and promoting additional inducible regulatory T cell differentiation (iTreg) [49], thus preventing uncontrolled inflammation and/or autoimmunity [50,51,52,53]. Rosa et al. (2012) [54] demonstrated that tissue levels of TGFβ-1 were increased in rats following EPA and DHA administration through fish oil. Similarly, increased TGF-β1 mRNA and protein secretion in colonic cell lines in response to commensal bacteria *Lactobacillus gasseri* was enhanced by pre-treatment with EPA [55]. This is in the line with present finding of enhanced capacity of activated PBMCs for TGFβ-1 secretion following consumption of *n*-3 PUFA-enriched eggs. Interestingly, levels of secreted TGFβ-1 negatively correlated with the frequency of peripheral nTreg cell population in the *n*-3 PUFAs group. Even though TGFβ-1 exhibits both anti- and pro-inflammatory properties [56,57], the latter is manifested only in combination with IL-6 (promotion of Th17 differentiation) [58] which was decreased in the *n*-3 PUFAs group. 

In healthy individuals, nTreg cells represent around 2–10% of the total T helper cells pool, with slightly lower frequencies found in peripheral blood [59,60]. Treg cells have shown noticeable therapeutic potential in terms of their expansion to control autoimmune and inflammatory disorders or, at the other side of the therapeutic spectra, their depletion to promote T effector cell function and eliminate cancer [59,61]. However, most recent studies reported their substantial phenotypic and functional variability beyond sole immunosuppression [62,63,64,65,66]. Decreased rates of peripheral Treg cells following *n*-3 PUFA-enriched functional food consumption in our study can be explained by the previously reported inhibitory effect of dietary DHA on both migratory and suppressive Treg cells functions which was proven as dose dependent in vitro and in vivo (100 µmol/L) [61,67]. Interestingly, a diet rich in *n*-3 PUFAs upregulates expression of Treg cell markers, TGFβ-1, and Foxp3 [61,67], which was confirmed by our results, and explains the negative correlation between TGFβ-1 supernatant concentration and Tregs.

## 5. Conclusions

The results of the present study have demonstrated the important role of *n*-3 PUFAs on immunoregulation when consumed in the form of functional foods. This is the first study of its kind to determine that *n*-3 PUFAs can modify key mediators of inflammation and alter the frequency of particular lymphocyte subpopulations in human subjects through modified foodstuff. Such a set of achieved conditions can lead to a shift towards systemic inflammation resolving environment in the population of young and healthy individuals.

## Figures and Tables

**Table 1:** General and biochemical characteristics of study population.

Parameter	Control Group	*n*-3 PUFA Group	*p*	Reference Range
*N* (W/M)	21 (10/11)	19 (10/9)	-	-
Age (years)	23.8 ± 2.79	23.8 ± 2.34	0.954	-
BMI (kg/m^2^)	24.2 ± 3.01	22.72 ± 3.53	0.168	18.5–24.9
Urea (mmol/L)	5.27 ± 1.32	5.91 ± 1.25	0.183	2.8–8.3
Creatinine (µmol/L)	78.85 ± 16.68	85.67 ± 18.29	0.289	49–90
Sodium (mmol/L)	138.1 ± 2.36	137.92 ± 1.51	0.812	137–146
Potassium (mmol/L)	4.15 ± 0.25	4.24 ± 0.23	0.283	3.9–5.1
Calcium (mmol/L)	2.44 ± 0.06	2.41 ± 0.07	0.265	2.14–2.53
Iron (µmol/L)	17.55 ± 5.96	19.34 ± 6.09	0.419	8.0–30.0
Transferrin (g/L)	2.87 ± 0.49	2.78 ± 0.39	0.62	2.00–3.60
Fasting blood glucose (mmol/L)	4.82 ± 0.57	4.64 ± 0.82	0.474	4.2–6.0
hsCRP (mg/L)	1.85 ± 2.41	1.53 ± 1.36	0.672	<5.00
Cholesterol (mmol/L)	5.26 ± 0.96	4.39 ± 0.74	0.012 *	<5.00
Triglycerides (mmol/L)	1.09 ± 0.48	1.25 ± 1.08	0.573	<1.70
HDL cholesterol (mmol/L)	1.61 ± 0.37	1.38 ± 0.29	0.071	>1.20
LDL cholesterol (mmol/L)	3.27 ± 0.81	2.71 ± 0.49	0.037 *	<3.00
Leukocytes (×10E9/L)	6.17 ± 1.38	6.12 ± 1.44	0.918	4.4–11.6
Platelets (×10E9/L)	256 ± 65.97	228.83 ± 36.82	0.202	178–420
Erythrocytes (×10E12/L)	4.77 ± 0.33	4.72 ± 0.39	0.692	4.07–5.42
Haemoglobin (g/L)	140.2 ± 10.99	142.75 ± 12.05	0.544	118–149
Haematocrit	0.41 ± 0.03	0.41 ± 0.03	0.702	0.354–0.450
MCV (fL)	86.11 ± 3.73	88.7 ± 3.15	0.054	76.5–92.1
MCH (pg)	29.45 ± 1.49	30.23 ± 1.12	0.126	24.3–31.5
MCHC (g/L)	341.85 ± 5.58	340.92 ± 6.36	0.667	304–346
RDW-CV (%)	13.8 ± 0.92	14.08 ± 0.51	0.35	9.0–15.0
MPV (fL)	10.43 ± 0.49	10.73 ± 0.58	0.129	7.0–10.4

Results are expressed as average ± standard deviation (SD). *N*—number of participants; W—women; M—men; BMI—body mass index; hsCRP—high-sensitivity C-reactive protein; HDL—high-density lipoprotein; LDL—low-density lipoprotein; MCV—mean corpuscular volume; MCH—mean corpuscular haemoglobin; MCH—mean corpuscular haemoglobin concentration; RDW-CV—red cell distribution width; MPV—mean platelet volume. Student’s *t*-test; significance level *p* < 0.05 * control group vs. *n*-3 PUFAs group. Reference range—general population.

## Data Availability

The data presented in this study are available on request from the corresponding author.

## Supplementary Materials

The following are available online at https://www.mdpi.com/article/10.3390/nu13061851/s1, Figure S1: CONSORT 2010 checklist of information to include when reporting a randomized trial.

## References

[1] Chen L., Deng H., Cui H., Fang J., Zuo Z., Deng J., Li Y., Wang X., Zhao L. (2017). Inflammatory responses and inflammation-associated diseases in organs. Oncotarget.

[2] Freire M.O., Van Dyke T.E. (2013). Natural resolution of inflammation. Periodontol. 2000.

[3] Cano R.L.E., Lopera H.D.E. (2013). Introduction to T and B lymphocytes. Autoimmunity: From Bench to Bedside.

[4] Charles A., Janeway J., Travers P., Walport M., Shlomchik M.J. (2001). General Properties of Armed Effector T Cells. Immunobiology: The Immune System in Health and Disease.

[5] Wambre E., James E.A., Kwok W.W. (2012). Characterization of CD4+ T cell subsets in allergy. Curr. Opin. Immunol..

[6] Chen X., Oppenheim J.J. (2014). Th17 cells and T _regs_: Unlikely allies. J. Leukoc. Biol..

[7] Allen M.J., Fan Y.Y., Monk J.M., Hou T.Y., Barhoumi R., McMurray D.N., Chapkin R.S. (2014). *n*–3 PUFAs Reduce T-Helper 17 Cell Differentiation by Decreasing Responsiveness to Interleukin-6 in Isolated Mouse Splenic CD4+ T Cells123. J. Nutr..

[8] Switzer K.C., McMurray D.N., Morris J.S., Chapkin R.S. (2003). (*n*-3) Polyunsaturated Fatty Acids Promote Activation-Induced Cell Death in Murine T Lymphocytes. J. Nutr..

[9] Araujo P., Belghit I., Aars N. (2019). The Effect of Omega-3 and Omega-6 Polyunsaturated Fatty Acids on the Production of Cyclooxygenase and Lipoxygenase Metabolites by Human Umbilical Vein Endothelial Cells. Nutrients.

[10] Fabian C., Kimler B., Hursting S. (2015). Omega-3 fatty acids for breast cancer prevention and survivorship. Breast Cancer Res. BCR.

[11] Abbas A.K., Lichtman A.H. (2011). Basic Immunology: Functions and Disorders of the Immune System.

[12] Ferreira V.L., Borba H.H.L., Bonetti A., Leonart L., Pontarolo R. (2018). Cytokines and Interferons: Types and Functions. Autoantibodies and Cytokines. https://www.intechopen.com/books/autoantibodies-and-cytokines/cytokines-and-interferons-types-and-functions.

[13] Foster J.R. (2001). The functions of cytokines and their uses in toxicology. Int. J. Exp. Pathol..

[14] Li C., Wu X., Liu S., Shen D., Zhu J., Liu K. (2020). Role of Resolvins in the Inflammatory Resolution of Neurological Diseases. Front. Pharmacol..

[15] Yang L.G., Song Z.X., Yin H., Wang Y.Y., Shu G.F., Lu H.X., Wang S.K., Sun G.J. (2016). Low *n*-6/n-3 PUFA Ratio Improves Lipid Metabolism, Inflammation, Oxidative Stress and Endothelial Function in Rats Using Plant Oils as *n*-3 Fatty Acid Source. Lipids.

[16] Wijendran V., Hayes K.C. (2004). Dietary n-6 and *n*-3 fatty acid balance and cardiovascular health. Annu. Rev. Nutr..

[17] Husted K.S., Bouzinova E.V. (2016). The importance of *n*-6/*n*-3 fatty acids ratio in the major depressive disorder. Medicina.

[18] Nindrea R.D., Aryandono T., Lazuardi L., Dwiprahasto I. (2019). Association of Dietary Intake Ratio of n-3/n-6 Polyunsaturated Fatty Acids with Breast Cancer Risk in Western and Asian Countries: A Meta-Analysis. Asian Pac. J. Cancer Prev..

[19] Jamilian M., Hashemi Dizaji S., Bahmani F., Taghizadeh M., Memarzadeh M.R., Karamali M., Akbari M., Asemi Z. (2017). A Randomized Controlled Clinical Trial Investigating the Effects of Omega-3 Fatty Acids and Vitamin E Co-Supplementation on Biomarkers of Oxidative Stress, Inflammation and Pregnancy Outcomes in Gestational Diabetes. Can. J. Diabetes.

[20] Miyata J., Arita M. (2015). Role of omega-3 fatty acids and their metabolites in asthma and allergic diseases. Allergol. Int..

[21] Stupin A., Mihalj M., Kolobarić N., Šušnjara P., Kolar L., Mihaljević Z., Matic A., Stupin M., Jukic I., Kralik Z. (2020). Anti-Inflammatory Potential of n-3 Polyunsaturated Fatty Acids Enriched Hen Eggs Consumption in Improving Microvascular Endothelial Function of Healthy Individuals—Clinical Trial. IJMS.

[22] Stupin M., Kibel A., Stupin A., Selthofer-Relatić K., Matić A., Mihalj M., Mihaljevic Z., Jukic I., Drenjancevic I. (2019). The Physiological Effect of n-3 Polyunsaturated Fatty Acids (*n*-3 PUFAs) Intake and Exercise on Hemorheology, Microvascular Function, and Physical Performance in Health and Cardiovascular Diseases; Is There an Interaction of Exercise and Dietary n-3 PUFA Intake?. Front Physiol..

[23] Agh F., Honarvar N.M., Djalali M., Nematipour E., Gholamhoseini S., Zarei M., Ansari S., Javanbakht M.H. (2017). Omega-3 Fatty Acid Could Increase One of Myokines in Male Patients with Coronary Artery Disease: A Randomized, Double-Blind, Placebo-Controlled Trial. Arch. Iran. Med..

[24] Saboori S., Koohdani F., Nematipour E., Yousefi Rad E., Saboor-Yaraghi A.A., Javanbakht M.H. (2016). Beneficial effects of omega-3 and vitamin E coadministration on gene expression of SIRT1 and PGC1α and serum antioxidant enzymes in patients with coronary artery disease. Nutr. Metab. Cardiovasc. Diseases.

[25] Haghiac M., Yang X., Presley L., Smith S., Dettelback S., Minium J., Belury M.A., Catalano P.M., Hauguel-de-Mouzon S. (2015). Dietary Omega-3 Fatty Acid Supplementation Reduces Inflammation in Obese Pregnant Women: A Randomized Double-Blind Controlled Clinical Trial. PLoS ONE.

[26] Azuma M.M., Gomes-Filho J.E., Ervolino E., de Barros Morais Cardoso C., Pipa C.B., Kawai T., Conti L.C., Cintra L.T.A. (2018). Omega-3 Fatty Acids Reduce Inflammation in Rat Apical Periodontitis. J. Endod..

[27] Jones M.L., Mark P.J., Mori T.A., Keelan J.A., Waddell B.J. (2013). Maternal Dietary Omega-3 Fatty Acid Supplementation Reduces Placental Oxidative Stress and Increases Fetal and Placental Growth in the Rat1. Biol. Reprod. Internet.

[28] Lluís L., Taltavull N., Muñoz-Cortés M., Sánchez-Martos V., Romeu M., Giralt M. (2013). Protective effect of the omega-3 polyunsaturated fatty acids: Eicosapentaenoic acid/Docosahexaenoic acid 1:1 ratio on cardiovascular disease risk markers in rats. Lipids Health Dis..

[29] McGuinness J., Neilan T.G., Sharkasi A., Bouchier-Hayes D., Redmond J.M. (2006). Myocardial protection using an omega-3 fatty acid infusion: Quantification and mechanism of action. J. Thorac. Cardiovasc. Surg..

[30] Drenjancevic I., Kralik G., Kralik Z., Mihalj M., Stupin A., Novak S., Grcevic M. (2017). Polyunsaturated Fatty Acids on Cardiovascular Health: Revealing Potentials of Functional Food.

[31] Ellulu M.S., Khaza’ai H., Patimah I., Rahmat A., Abed Y. (2016). Effect of long chain omega-3 polyunsaturated fatty acids on inflammation and metabolic markers in hypertensive and/or diabetic obese adults: A randomized controlled trial. Food Nutr. Res..

[32] Gibney M.J., Lanham-New S.A., Cassidy A., Vorster H.H. (2002). Introduction to Human Nutrition.

[33] Kolobarić N., Gradinjan Centner M., Šušnjara P., Matić A., Drenjančević I. (2020). Anthropometric and Biochemical Parameters in Relation to Dietary Habits as Early Indicator of Cardiovascular Impairment in Young Adult Cohort. Int. J. Environ. Res. Public Health.

[34] Benistant C., Achard F., Marcelon G., Lagarde M. (1993). Platelet inhibitory functions of aortic endothelial cells. Effects of eicosapentaenoic and docosahexaenoic acids. Atherosclerosis.

[35] Hadjiagapiou C., Kaduce T.L., Spector A.A. (1986). Eicosapentaenoic acid utilization by bovine aortic endothelial cells: Effects on prostacyclin production. Biochim. Biophys. Acta (BBA) Lipids Lipid Metab..

[36] Gu Z., Shan K., Chen H., Chen Y.Q. (2015). n-3 Polyunsaturated Fatty Acids and Their Role in Cancer Chemoprevention. Curr. Pharmacol. Rep..

[37] Luis D., Conde R., Aller R., Izaola O., González Sagrado M., Pérez-Castrillón J., Duenas A., Romero E. (2009). Effect of omega-3 fatty-acids on cardiovascular risk factors in patients with type 2 diabetes mellitus and hypertriglyceridemia: An open study. Eur. Rev. Med. Pharmacol. Sci..

[38] Mansara P., Ketkar M., Deshpande R., Chaudhary A., Shinde K., Kaul-Ghanekar R. (2015). Improved antioxidant status by omega-3 fatty acid supplementation in breast cancer patients undergoing chemotherapy: A case series. J. Med. Case Rep..

[39] Malkowski M.G., Thuresson E.D., Lakkides K.M., Rieke C.J., Micielli R., Smith W.L., Garavito R.M. (2001). Structure of Eicosapentaenoic and Linoleic Acids in the Cyclooxygenase Site of Prostaglandin Endoperoxide H Synthase-1. J. Biol. Chem..

[40] Brossard N., Croset M., Pachiaudi C., Riou J.P., Tayot J.L., Lagarde M. (1996). Retroconversion and metabolism of [13C]22:6n-3 in humans and rats after intake of a single dose of [13C]22:6n-3-triacylglycerols. Am. J. Clin. Nutr..

[41] Higdon J. (2003). Essential Fatty Acids. Linus Pauling Institute. https://lpi.oregonstate.edu/mic/other-nutrients/essential-fatty-acids.

[42] Serhan C.N., Levy B.D. (2018). Resolvins in inflammation: Emergence of the pro-resolving superfamily of mediators. J. Clin. Investig..

[43] Haworth O., Cernadas M., Yang R., Serhan C.N., Levy B.D. (2008). Resolvin E1 regulates interleukin-23, interferon-γ and lipoxin A4 to promote resolution of allergic airway inflammation. Nat. Immunol..

[44] Levy B.D. (2012). Resolvin D1 and Resolvin E1 Promote the Resolution of Allergic Airway Inflammation via Shared and Distinct Molecular Counter-Regulatory Pathways. Front Immunol..

[45] Layé S., Madore C., St-Amour I., Delpech J.C., Joffre C., Nadjar A., Calon F. (2015). N-3 polyunsaturated fatty acid and neuroinflammation in aging and Alzheimer’s disease. NUA.

[46] Schwanke R.C., Marcon R., Bento A.F., Calixto J.B. (2016). EPA- and DHA-derived resolvins’ actions in inflammatory bowel disease. Eur. J. Pharmacol..

[47] Zhang J.-M., An J. (2007). Cytokines, Inflammation and Pain. Int. Anesthesiol. Clin..

[48] Lee J.-E., Sun Y., Gjorstrup P., Pearlman E. (2015). Inhibition of Corneal Inflammation by the Resolvin E1. Investig. Ophthalmol. Vis. Sci..

[49] Hsu P., Santner-Nanan B., Hu M., Skarratt K., Lee C.H., Stormon M., Wong M., Fuller S.J., Nanan R. (2015). IL-10 Potentiates Differentiation of Human Induced Regulatory T Cells via STAT3 and Foxo1. J. Immunol..

[50] Chen W., Jin W., Hardegen N., Lei K.-J., Li L., Marinos N., McGrady G., Wahl S.M. (2003). Conversion of peripheral CD4+CD25- naive T cells to CD4+CD25+ regulatory T cells by TGF-beta induction of transcription factor Foxp3. J. Exp. Med..

[51] Konkel J.E., Jin W., Abbatiello B., Grainger J.R., Chen W. (2014). Thymocyte apoptosis drives the intrathymic generation of regulatory T cells. Proc. Natl. Acad. Sci. USA.

[52] Levings M.K., Bacchetta R., Schulz U., Roncarolo M.G. (2002). The role of IL-10 and TGF-beta in the differentiation and effector function of T regulatory cells. Int. Arch. Allergy Immunol..

[53] Szondy Z., Sarang Z., Kiss B., Garabuczi É., Köröskényi K. (2017). Anti-inflammatory Mechanisms Triggered by Apoptotic Cells during Their Clearance. Front Immunol..

[54] Rosa D.D., Lourenço F.C., da Fonseca A.C.M., de Sales R.L., Ribeiro S.M.R., Neves C.A., Peluzio M.d.C.G. (2012). Fish Oil Improves the Lipid Profile and Reduces Inflammatory Cytokines in Wistar Rats With Precancerous Colon Lesions. Nutr. Cancer.

[55] Bentley-Hewitt K.L., De Guzman C.E., Ansell J., Mandimika T., Narbad A., Lund E.K. (2014). Polyunsaturated fatty acids modify expression of TGF-β in a co-culture model ultilising human colorectal cells and human peripheral blood mononuclear cells exposed to Lactobacillus gasseri, Escherichia coli and Staphylococcus aureus. Eur. J. Lipid Sci. Technol..

[56] Han G., Li F., Singh T.P., Wolf P., Wang X.-J. (2012). The Pro-inflammatory Role of TGFβ1: A Paradox?. Int. J. Biol. Sci..

[57] Wan Y.Y., Flavell R.A. (2007). ‘Yin-Yang’ functions of TGF-β and Tregs in immune regulation. Immunol. Rev..

[58] Oukka M. (2007). Interplay between pathogenic Th17 and regulatory T cells. Ann. Rheum. Dis..

[59] Niedźwiecki M., Budziło O., Adamkiewicz-Drożyńska E., Pawlik-Gwozdecka D., Zieliński M., Maciejka-Kembłowska L., Szczepański T., Trzonkowski P. (2019). CD4+CD25highCD127low/-FoxP3+ Regulatory T-Cell Population in Acute Leukemias: A Review of the Literature. J. Immunol. Res..

[60] Sakaguchi S., Kassiotis G., Liston A. (2011). Regulatory T Cells: History and Perspective. Regulatory T Cells: Methods and Protocols.

[61] Yessoufou A., Plé A., Moutairou K., Hichami A., Khan N.A. (2009). Docosahexaenoic acid reduces suppressive and migratory functions of CD4CD25 regulatory T-cells. J. Lipid Res..

[62] Černý V., Hrdý J., Novotná O., Petrásková P., Boráková K., Kolářová L., Prokešová L. (2018). Distinct characteristics of Tregs of newborns of healthy and allergic mothers. PLoS ONE.

[63] Jung M.K., Kwak J.-E., Shin E.-C. (2017). IL-17A-Producing Foxp3 + Regulatory T Cells and Human Diseases. Immune Netw..

[64] Khoshmirsafa M., Seif F., Bagheri N., Beshkar P., Mousavi M., Shirzad H. (2018). Correlation of interleukin 6 and transforming growth factor β1 with peripheral blood regulatory T cells in rheumatoid arthritis patients: A potential biomarker. Cent Eur. J. Immunol..

[65] Sawant D.V., Yano H., Chikina M., Zhang Q., Liao M., Liu C., Callahan D.J., Sun Z., Sun T., Tabib T. (2019). Adaptive plasticity of IL-10 + and IL-35 + T reg cells cooperatively promotes tumor T cell exhaustion. Nat. Immunol..

[66] Shevach E.M. (2009). Mechanisms of foxp3+ T regulatory cell-mediated suppression. Immunity.

[67] Issazadeh-Navikas S., Teimer R., Bockermann R. (2011). Influence of Dietary Components on Regulatory T Cells. Mol. Med..

